# A review of osteoporotic vertebral fracture animal models

**DOI:** 10.1186/s12938-025-01372-x

**Published:** 2025-04-07

**Authors:** Zhichao Qi, Guozhu Ye, Zhiyi Liu, Jie Zhang, Weidong Xie, Yu Li, Wenbin Yang

**Affiliations:** 1 The Department of Orthopaedics, Guangdong Provincial Second Hospital of Traditional Chinese Medicine, No.60, Heng Fu Lu, Guangzhou, 510095 China; 2 The Fifth Clinical College of Guangzhou University of Chinese Medicine, Guangzhou, China; 3 Department of Orthopaedic Surgery, Hirosaki University, No.53 Honcho, Aomori, Japan

**Keywords:** Animal models, Vertebral fracture, Osteoporosis

## Abstract

**Introduction:**

Osteoporotic vertebral fractures are a common outcome of osteoporosis, imposing a substantial economic burden. The development of reliable animal models is essential for advancing research. This review examines osteoporotic vertebral fracture models across various animal species.

**Methods:**

The review compares and analyzes the different approaches used to model osteoporotic vertebral fractures in experimental animals, synthesizing the existing design protocols.

**Results:**

Rats and sheep are the primary experimental animals utilized in vertebral fracture research. The predominant approach in model design remains the creation of bone defects to simulate vertebral fractures. The spontaneous fracture model is primarily applicable to small species, such as transgenic mice. Rabbits and zebrafish are not suitable for modeling vertebral fractures due to the low cancellous bone content in their lumbar. The bone loss in the lumbar cancellous bone of the dog osteoporosis model is minimal, making it unsuitable for fracture modeling.

**Conclusions:**

The bone defect model remains the most widely used approach for osteoporotic vertebral fractures. However, the stress compression model shows promise as a potential focal point for future investigations.

## Introduction

Osteoporosis is a systemic skeletal disorder characterized by diminished bone mass, deterioration of bone microstructure, and increased bone fragility, significantly impacting the quality of life and imposing a substantial economic burden [1]. Osteoporotic fractures are the primary outcome of this condition. Notably, the majority of osteoporotic fractures occur in metaphyseal regions due to a significant reduction in bone mineral density (BMD), such as the distal radius, proximal humerus, proximal femur or vertebral bodies, rather than in the diaphyseal areas of long bones [2].

Osteoporotic animal models are essential research method for studying and treating human osteoporosis in the field of medicine. Human osteoporosis is one of the major age-related diseases that encompasses several primary forms, including postmenopausal osteoporosis, disused osteoporosis, and glucocorticoid-induced osteoporosis. The design of osteoporotic animal models is also informed by the known forms of human osteoporosis [3]. The large skeleton structure and complex Haversian system of humans are major challenges in osteoporotic fracture animal models.

Human osteoporotic fractures most commonly occur in the hip, followed by the vertebral body. The patient’s overall remaining lifetime risk of osteoporotic vertebral fractures (20.6%) is second only to that of osteoporotic hip fractures (23.5%) [4]. The metaphyseal osteoporotic fracture animal model of long bones is extensively utilized and studied due to its operational simplicity and high reproducibility [5]. Despite the severity of osteoporotic vertebral fractures being surpassed only by osteoporotic hip fractures in humans, research on osteoporotic vertebral fracture animal models is less prominent compared to long bone osteoporotic metaphyseal fracture animal models. It is important to note that while both vertebrae and long bone epiphyses are metaphyseal regions, they are not identical [6]. This review aims to provide a comprehensive overview of existing osteoporotic vertebral fracture animal models to facilitate further clinical learning and research.

## Materials and methods

This review focuses on in vivo animal models of osteoporotic vertebral fractures, with no specific limitations regarding osteoporosis modeling. We included studies with in vivo vertebral fracture modeling, and excluded studies that only performed ex vivo mechanical compression experiments after establishing osteoporosis and euthanizing animals. Our objective was to summarize data on in vivo fracture models rather than those based on in vitro mechanical experiments.

The review included several commonly used animals in osteoporosis models and fracture models, such as rats, mice, rabbits, sheep, and dogs, as well as some species with limited applications, including zebrafish, pigs, and rhesus monkeys [3, 7, 8]. During the search process, it became apparent that osteoporotic animal models were more commonly utilized than vertebral fracture models. Consequently, we included studies that primarily focused on vertebral fracture models without explicitly modeling osteoporosis due to their methodological significance. When multiple osteoporosis modeling methods exist within the same species, we prioritize those that are universal and more helpful for modeling vertebral fractures.

A literature search was performed using the MEDLINE database, provided by the U.S. National Library of Medicine, with results limited to publications up to December 2025. The search utilized the following terms: “osteoporosis animal models”, “rat”, “mouse/mice”, “rabbit”, “sheep”, “zebrafish”, “dog/canine”, “monkey”, “pig”, “vertebral fracture”, “osteoporotic vertebrae”, “bone defect”, “spine/spinal fracture”, “spine/spinal”, and “vertebral defect”. Additionally, a comprehensive manual search was conducted through the references cited in the included papers. Research literature on osteoporotic vertebral fracture model were categorized by animal species (Table 1).

**Table 1:** Summary of retrieved articles

Author	Animal	Osteoporotic methods	Bone	Vertebral fracture methods	Application
Wang et al. [13]	Female SD rats, 8 weeks old	OVX + 3 months LCD (0.01% calcium, 0.77% phosphate)	Caudal vertebrae	Spontaneous low bone mass and microcracks	CaSO_4_ cement filling
Shen et al. [9]	Female SD rats, 6 months old	OVX for 3 months	Lumbar vertebrae	Hemispheric bone defect (3 mm) in L6	Introduce to the new model
Sakata et al. [10, 11]	Female SD rats, 12 weeks old	OVX for 8 weeks	Lumbar vertebrae	Hemispheric bone defect (4 mm) in L3	Introduce to the new model
Makino et al. [16]	Female SD rats, 8 weeks old	OVX for 4 weeks		Cylindrical bone defect (1.5 mm × 3 mm) in L4, L5 and L6	Abaloparatide promotes bone repair
Shapiro et al. [12]	Female nude rats, 6 weeks old	OVX + 4 months LCD (0.01% calcium, 0.77% phosphate)	Lumbar vertebrae	Cylindrical bone defect (2 mm × 5 mm) in L4 and L5	Introduce to the new model
Geoffroy et al. [21]	Transgenic mice	Overexpressing core-binding factor alpha 1 (Cbfa1)	Multiple sites	Spontaneous fractures	Introduce to the new model
Geoffroy et al. [22]	Transgenic mice	Overexpressing core-binding factor alpha 1 (Cbfa1)	Multiple sites	Spontaneous fractures	Strontium ranelate therapy for fractures
Wang et al. [28]	Male/female New Zealand rabbit, 2 months old	No	Lumbar vertebrae	Bone defect (4 mm × 3 mm × 3 mm) in L3	Composite bone cement for repairing bone defects
Galovich et al. [35]	Merino female sheep, 4–6 years old	OVX + LCD (1.5 g calcium and 100 IU vitamin D3/day) + injected with methylprednisolone (O + D + S) for 7 months	Lumbar vertebrae	Cylindrical bone defect (5 mm × 10 mm) in L1, L2, L3, L4, L5 and L6	Analyses of calcium phosphate cement with polymethylmethacrylate
Verron et al. [36]	Adult female sheep	OVX for 6 months	Lumbar vertebrae	Bone defect (8 mm × 10 mm × 20 mm) in L3	Analyses of bisphosphonate-loaded calcium phosphate cement filling
Bungartz et al. [37]	Merino female sheep, 6–9 years old	Aged	Lumbar vertebrae	Cylindrical bone defect (5 mm × 14 mm) in L4 and L5	GDF5 augments the bone formation
Phillips et al. [43]	Adult female sheep	OVX + LCD for 6 months	Lumbar vertebrae	Cylindrical bone defect (8 mm × 5 mm) in L3 and L4	The enhancing effect of BMP-7 in vivo
Gunnella et al. [44]	Merino female sheep, 6–9 years old	Aged	Lumbar vertebrae	Cylindrical bone defect (5 mm × 14 mm) in L4 and L5	Low-dose BMP-2 enhance the bone formation
Eschler a et al. [39]	Female Merino sheep, 5 years old	OVX + LCD (1.6 g calcium, 2.6 g phosphorus, 183 IU vitamin D3)	Lumbar vertebrae	Stress compression fracture	Introduce to the new model
Bohns et al. [50]	Both sexes, wild-type AB strain zebrafish, 7 months old	Prednisolone + dimethyl sulfoxide in water for 21 days, concentration of 50 μM	Spine	Spontaneous vertebral bone mass reduction	Alendronate improved bone mass
Li et al. [45]	Both sexes, wild-type AB strain zebrafish, 6 months old	Dexamethasone 21-phosphate in water for 7 days, concentration of 25 μM	Fin	Fish tail fin stress fracture	Explore the osteogenic potential of chitosan–quercetin bio-conjugate
Turner et al. [52]	Mature large hounds	No	Lumbar vertebrae	Bone defect (18 mm × 5 mm × 22 mm) in L1, L3	Vertebroplasty compare calcium phosphate cement with polymethylmethacrylate
Oshima et al. [53]	Beagles, 6 months old	No	Lumbar vertebrae	Bone defect (2 mm × 2 mm × 3.6 mm)	Evaluation of vertebroplasty using hydroxyapatite blocks
Shao et al. [54]	Rhesus monkeys, 5–7 years old	No	Lumbar vertebrae	Bone defect (18 mm × 5 mm × 22 mm) in L4	Hydrogel containing BMP-2 and BMSCs on lumbar vertebral defect repair
Pelled et al. [55]	Minipigs	No	Lumbar vertebrae	Cylindrical bone defect (4 mm × 15 mm) in lumbar vertebrae	BMP6-engineered MSCs induce vertebral bone repair

*OVX* ovariectomy, *SD rats* Sprague–Dawley rats, *LCD* low calcium diet

## Results

### Rat model

Shen et al. [9] utilized ovariectomy (OVX) to create osteoporosis models in 6-month-old female Sprague–Dawley (SD) rats. Three months after OVX, osteoporotic rats were anesthetized and exposed via an anterior midline transperitoneal approach. Researchers used an electric drill with a 3-mm-diameter drill creating an appropriately 3-mm-diameter hemispheric defect through the anterior part of the rat vertebra L6 after removing anterior longitudinal ligament and periosteum. The BMD of osteoporotic group was significantly lower than that of the control group, and the bone defect of L6 was never fully repaired after 16 weeks. Sakata et al. [10, 11]. Utilized OVX to modeling osteoporosis in 12-week-old female SD rats. Eight weeks after OVX, an electric drill with a 4-mm-diameter drill was used to create a hemispheric 4 mm bone defect in the center of the anterior L3 in osteoporotic rats. After confirming that vertebral bone defects can persist for 12 weeks without repair, the researchers further investigated how implanted materials can facilitate bone regeneration.

Shapiro et al. [12] replicated bone defect method in 6-week-old nude rats (athymic rats). Nude rats were fed with low calcium diet (LCD, 0.01% calcium, 0.77% phosphate) on 4 months after OVX. An electric drill with a 2-mm-diameter drill was used to create a 5-mm-deep bone defect in the center of the anterior L4 via an anterior midline transperitoneal approach in osteoporotic nude rats. But the cylinders they drilled were relatively small a resulting in partial repair of the defect after 4 weeks modeling.

Wang et al. [13] induced osteoporosis in 8-week-old female SD rats with a LCD (0.01% calcium, 0.77% phosphate) for 3 months after OVX, which resulted in fractures and fissures in the coccygeal vertebrae under natural conditions. They filled CaSO_4_ cement into the coccygeal vertebrae to observe resorption. Their findings indicate that the caudal vertebrae exhibit pathological bone changes consistent with osteoporosis, including decreased BMD, reduced trabecular network density, cortical thinning, and diminished mechanical strength.

Rats were widely used in osteoporosis research due to their cost-effectiveness, short growth cycle, and ease of handling. Rats enter menopause until 18–24 months of age, becoming aging rats, and few modeled osteoporotic rats experience natural menopause. OVX is the most widely utilized method for modeling osteoporosis in rats [14]. Combining OVX with the LCD (0.01% calcium, 0.77% phosphate) may result in more significant effects. Six-months-old SD and Wistar rats are most commonly strains due to similar responses to OVX. After OVX surgery, estrogen levels in rats decreased significantly, leading to increased bone resorption and disrupted bone metabolism especially cancellous bone. Femoral neck (70%), lumbar vertebral (40%), and proximal tibia (30%), characterized by their higher cancellous bone, are the most suitable sites for fracture modeling. The earliest observed instances of bone loss occurred at 30 days, 60 days, and 14 days, respectively, indicating that bone resorption exceeded bone formation. OVX does not induce bone distal tibia metaphysis and caudal vertebrae. Rats’ vertebrae are very similar width-to-depth axial aspect ratios to human vertebrae in the lumbar regions, especially the L2–L5 vertebrae, but rat vertebrae are more slender than human vertebrae [15].

One of the primary limitations of the rat skeletal system is its incomplete Haversian system. The Haversian system, a network of microscopic canals that house blood vessels and nerves, is unevenly distributed in rats, with greater density observed on the medullary side of the cortical bone and sparse distribution on the cortical side [16]. This uneven distribution contributes to the unique characteristics of bone remodeling of rats after OVX, including the presence of longitudinal bone growth in mature rats, less cortical bone loss, higher modeling activity, and absence of naturally fragility fracture in the rat skeleton [3, 7, 14]. Bone defects are typically created in shapes such as square, cylindrical, or circular, with a recommended minimum defect diameter of 3 mm [17, 18]. Researchers often use spherical drill bits to directly penetrate cortical bone at the anterior edge of the vertebra for creating bone defects. A 3-mm vertebral bone defect of OVX rats typically remains open 12 to 16 weeks after OVX before natural repair begins. If bone conditions allow, the minimum length of bone defects should exceed than 3 mm [18]. Defects smaller than 3 mm often heal within 4 weeks, even in osteoporotic models [12, 19, 20].

Based on the characteristics of the OVX model and the bone defect model, Shen et al. [9] proposed method should be more appropriate. We maintain a cautious and skeptical perspective towards Wang et al. [13] used the coccygeal vertebra modeling method according to the characteristics of OVX. This still requires sufficient evidence to prove the coccygeal vertebra model does meet the requirements for extensive research on bone repair and biomaterial implantation. Although the vertebral bone defect model has been widely used in rats, this model does not fully replicate the characteristics of vertebral compression fractures.

### Mouse model

Geoffroy et al. [21, 22] observed that transgenic mice overexpressing core-binding factor alpha 1 (Cbfa1) exhibited severe bone loss and multiple spontaneous fractures over a period of to 16 months. Cbfa1, a runt family transcription factor was identified as a transcriptional activator of osteoblast differentiation and has been proposed to be a master gene for bone. At 1 week of age, the transgenic mice exhibited temporarily normal bone mass. The BMD of the transgenic mice was significantly lower than that of the mice, showing a reduction of 37% at 2 months and 57% at 16 weeks. The fractures of the hindlimb bones and tail vertebrae in transgenic mice were more significant than those of the forelimbs at 16 months.

The primary methods for inducing osteoporosis in mice include OVX, suspension of disused bone, glucocorticoid induction, aging models, and targeted gene knockout [3, 23–26]. The common mice strains are C57BL/6 mice, ICR mice, and Swiss Webster mice. OVX developed earlier osteoporosis in mice than in rats. Disused osteoporosis is characterized by the principle that bone resorption exceeds bone formation under unstressed conditions. To observe this, lift the tail end of the mouse while keeping a head-down tilt at approximately 30 degrees. For 2 weeks, bone formation is mildly inhibited and bone resorption is markedly enhanced in C57BL/6 mice. High dose of prednisolone and dexamethasone were used to inducing osteoporosis in C57BL/6 mice, ICR mice, and Swiss Webster mice. A major problem in rodent models of glucocorticoid-induced osteoporosis is that cancellous bone is mainly affected in humans, but the loss of cancellous bone is not consistently observed in rodents. Genetic engineering technology is an alternative approach for developing an osteoporosis model. Genes related to osteoporosis modeling include estrogen receptor, ovarian development, aging, and bone cell metabolism.

Unfortunately, the various osteoporosis models in mice did not match sufficiently vertebral fracture models. Mice smaller size limits their capacity for bone mass acquisition and blood sample collection. Moreover, frequent surgical interventions in modeling may increase mortality risk in mice. Considering the feasibility and effectiveness of modeling fractures, the osteoporotic fracture models are more appropriate for long bones than for the vertebrae in mice [27]. Compared to spontaneous fractures, the bone defect model is less suitable for mice. However, the high cost and strong specificity of genetically modified mice restrict their potential for widespread application.

### Rabbit model

Wang et al. [28] created a 4 mm × 3 mm × 3 mm bone defect on the anterior of L3 vertebra of 2-month-old non-osteoporotic New Zealand white rabbits using a Lamina rongeur via a dorsal approach to evaluate the filling effect of a bone matrix gelatin composite cement. There remained a gap between the implant material and the bone defect at the fourth week. At the eighth week, the gap had become less distinct. At the twelfth week, bone tissue had integrated with the implant material. Zhang et al. [29] utilized adult New Zealand white female rabbits, aged 5 to 7 months, modeling osteoporosis. The rabbits were given methylprednisolone 1 mg/kg/d via intramuscular injection for 4 weeks after OVX. Zhu et al. [30] used 5-month-old New Zealand white female rabbits given intramuscularly dexamethasone 0.6 mg/kg/d after OVX for modeling osteoporosis.

Rabbits achieve complete skeletal maturity at 6–8 months. Adult rabbits possess a well-developed Haversian system, which is crucial for modeling cortical bone. Haversian system supports high bone turnover and predominant remodeling, making rabbits suitable for investigating the effects of anabolic agents [31]. Furthermore, rabbits are cost-effective and easy to house [32]. Compared with rats, rabbits have larger bones, which allows for easier collection of serum samples, making them more suitable for osteoporotic fracture treatment studies. However, despite these advantages, rabbits have less cancellous bone, which can complicate bone densitometry assessments [33]. Due to the unique hourglass-shaped structure of the lumbar vertebrae in rabbits, the proportion of lumbar cancellous bone in rabbits is lower than that in mice, rats, sheep, and dogs [34].

Alone OVX or glucocorticoid-induced may be insufficient for establishing osteoporosis models in rabbits. Combing OVX with glucocorticoid 0.5–1 mg/kg/d for a minimum of 4 weeks is a highly suitable approach. If the glucocorticoid dosage is less than 0.5 mg/kg/day, it is unlikely to significantly impact bone metabolism. Conversely, if the dosage exceeds 2 mg/kg/day, there is a substantially increased risk of mortality. It is essential to prepare the LCD for rabbits during the modeling process. The amount of calcium used in the rabbits’ LCD ranged from 0.07 to 0.15%, taking into account that a normal maintenance diet for rabbits has approximately 0.85% of calcium [33].

The osteoporotic vertebral fracture model in rabbits has received less attention compared with those in rats and sheep. In contrast to vertebrae, researchers more frequently utilize rabbit limb bones as in vivo fracture models. The method of simulating fractures with bone defects remains one of the few available techniques for creating vertebral fracture models. The new rabbit model can be developed based on OVX combined with glucocorticoids and the LCD, utilizing the vertebral bone defect model [28] methodology. Nevertheless, it is questionable whether the new model is suitable for the experimental conditions, as the rabbit vertebral body contains less cancellous bone.

### Sheep model

Galovich et al. [35] proposed scheme involved using 4- to 6-year-old female sheep that would be subjected to a LCD and a steroid regimen for 7 months after OVX to effectively model osteoporosis (O + D + S). The LCD was 1.5 g calcium and 100 IU vitamin D3 per day. The steroid regimen was intramuscular injections of 54 mg dexamethasone weekly. The researcher utilized a 5-mm drill to create holes that penetrated from one side of the vertebral body to other in L1, L2, L4, and L5, establishing bone defect models. This hole was subsequently filled with biocement to observe bone stability. Verron et al. [36] only utilized OVX for modeling osteoporosis in adult female sheep. A 8 mm high × 10 mm deep × 20 mm long bone defect was created into the L3 and L4 vertebral bodies with drills via a retroperitoneal approach following 6 months OVX. Some researchers utilized sheep with age-related bone mass loss as model for osteoporosis [37, 38]. The bone defect model, the minimum width at 5 mm, was created to evaluate the filling material.

Eschler et al. [39] introduced a modeling approach more applicable to clinical scenarios, specifically for vertebral body compression using orthopedic surgical instruments. In their research, osteoporosis in sheep was stablished 5.5 months following OVX, weekly corticosteroid administration (1.3 mg/kg dexamethasone), and the LCD in calcium (1.6 g), phosphorus (2.6 g), and vitamin D (183 IU). Subsequently, two 4.5 mm self-tapping cortical screws were inserted 5 mm below the upper/lower deck plates of L1 and L3. Jungbluth forceps were tightened to induce L2 compression fracture [39]. This method effectively replicates clinical conditions compared to the bone defect model, which preserves bone and surrounding tissues rather than complete removal. However, it does not eliminate complications such as infections and severe gastrointestinal side effects, which have necessitated euthanasia in some sheep [40].

Merino sheep are large animals with extended lifespan and abundant blood and bone tissue, making them suitable experimental subjects. Their skeletal structure is similar to that of humans, including woven bone, lamellar bone, and Haversian systems. Notably, the relevance of biochemical bone markers, such as alkaline phosphatase, osteocalcin, and crosslinks, can also be observed in sheep [38]. Sheep exhibit significant seasonal variation in BMD, characterized by lower bone mass during winter and higher bone mass during summer. Compared to humans, the lumbar cancellous bone of sheep exhibits higher microarchitectural indices, more densely packed bone trabeculae, lower porosity, and higher bone mass [34]. These characteristics form the basis of establishing an ideal fracture model. The simple OVX-induced osteoporosis model has a limited effect in sheep and cannot consistently impact bone mass and structure [38, 41, 42]. A combined induction protocol known as O + D + S can create a more stable model for osteoporosis. OVX was recommended performing in 5–6-year-old female sheep (O). The LCD was recommended containing at least one-third less calcium than the normal dosage, one-half less vitamin D than the normal dosage, and one-half less phosphorus than the normal dosage (D). Diverse steroid regimens were used for 5–6 months, such as daily injections of 0.6 mg/kg prednisolone, daily injections of 15–25 mg/kg methylprednisolone, 500 mg methylprednisolone every 3 weeks, 54 mg dexamethasone weekly, and so on (S).

The methodology of bone defects is most commonly applied in vertebral fracture modeling in sheep [35–37, 43, 44]. To inhibit rapid fracture healing post-modeling, the minimum edge length must be 5 mm. The bone defect model, due to its established nature, has become the standard approach in sheep models, particularly in evaluating surgical interventions and efficacy assessments. Nevertheless, this does not necessarily indicate that it is the most appropriate choice for every research. Among various bone defect models, the compression fracture model is particularly noteworthy for its pioneering role. The preservation of both bone and surrounding soft tissue at the fracture site makes it an invaluable tool for future research.

### Zebrafish model

Zebrafish and mammalian bones exhibits a high degree of similarity and a high conservation, including nearly all the bones matching, cells and mechanisms controlling skeletogenesis, early formation of the cartilaginous anlage and its replacement by bone, through endochondral and perichondral ossification, and the dermal ossification processes [45–47]. Zebrafish other features, such as a high number of offspring, a short generation time, external development, and translucent early life stages, have contributed to the popularity in bone studies. The trabecular bone structure of the zebrafish hourglass vertebral body is extremely thin, with a thickness at the micron level [48]. There were various methods to induce zebrafish osteoporosis model, including glucocorticoid induced osteoporosis, high glucose and high fat induced osteoporosis, iron overload induced osteoporosis, parathyroid hormone induced osteoporosis, botulinum toxin induced osteoporosis, and microgravity induced osteoporosis [49].

The conventional osteoporosis model in zebrafish is typically achieved through glucocorticoid induction. Bohns et al. [50] demonstrated this by administering prednisolone to the water tank (a final concentration of 50 μM) of 7-month-old zebrafish to induce glucocorticoid-induced osteoporosis. After 21 days, microfocus scanning of the whole vertebrae revealed a decrease in BMD and loss of mineralization. In comparison with vertebral models, fin and scale defect models in zebrafish offer a more accessible approach for investigating mineralization processes following fractures [45]. A key point highlighted is that the fin and scale of zebrafish is easier to be affected by glucocorticoids induced bone loss than that of spine [51]. Iron overload induced osteoporosis and microgravity induced osteoporosis can significantly lead to bone loss in the spine; however, the modeling costs associated with these conditions are considerably higher than those of glucocorticoid-induced osteoporosis, particularly microgravity-induced osteoporosis.

While zebrafish possess remarkable vertebral structures, a standardized method for implementing osteoporotic vertebral fracture models was not established. Moreover, even if a suitable model for osteoporotic vertebral fractures were developed, the small size and aquatic nature of zebrafish present considerable challenges for interventional surgery and treatment.

### Other large animal models

Turner et al. [52] created a defect measuring 18 × 5 × 22 mm on the vertebrae of skeletally mature large hounds to analyze the biomechanics of vertebroplasty without modeling osteoporosis. Oshima et al. [53] simulated vertebral fractures in small hunting dogs by drilling five 3-mm-diameter holes in the vertebral bodies and filling them with hydroxyapatite to investigate the stability following fracture repair in a non-osteoporosis model. This approach of using bone defects to study fracture healing has also been applied in rhesus monkeys [54] and pig [55] in non-osteoporosis models.

Large animals, including dogs, pigs, and monkeys, possess hormone metabolism and skeletal systems that more closely resemble those of humans [56]. Dogs are closely related to human life as companion animals deserves greater attention. Canine bone compositions are similar to humans, however, they can withstand greater compressive forces [57]. The structure and proportion of cancellous bone in dog lumbar vertebrae closely resemble those of humans, making them suitable for surgical intervention and modeling [34]. It is important to note that OVX alone will not create stable and effective osteoporosis models in dogs [56, 58]. Even when the LCD is added based on OVX to establish a relatively stable osteoporosis model in dogs, the most susceptible to bone are the jaw, skull, and ribs, rather than the vertebrae and long bones. After being affected by prednisone for 29 months, the spinal bone mass decreased slightly in dog osteoporosis model, but about 30% of dogs still had tolerance to prednisone [59]. Only sheep are more commonly employed in large animal models of osteoporotic fractures; research involving other large animals remains relatively limited.

## Discussion

We have reviewed animal models of osteoporotic vertebral fractures in various animals such as rats, mice, rabbits, and so on. We conducted the analyses based on animal characteristics, modeling vertebral fracture, and modeling osteoporosis. According to the review results, rats and sheep are the most suitable animals for modeling osteoporotic vertebral fractures. We listed some key points based on the universality and effectiveness of modeling.

Female 6-month-old SD rats are fed the LCD (0.01% calcium, 0.77% phosphate) for 3 months after OVX, leading to the osteoporotic model with severe bone loss. Using an electric drill creates a bone defect through the anterior part of the rat vertebra. The rat L1–L6 can meet the modeling requirements. The shape of the L2–L5 vertebrae closely resembles that of human lumbar vertebrae, making it more suitable for modeling. To prevent premature repair of the bone defect, the minimum diameter or width of the defect should be 3 mm.

The O + D + S protocol can be utilized to establish a stable osteoporotic model in sheep. Female 4- to 6-year-old merino sheep are fed the LCD for 6 months after OVX. The LCD was recommended containing at least one-third less calcium than the normal dosage, one-half less vitamin D than the normal dosage, and one-half less phosphorus than the normal dosage. Steroid, such as prednisolone, methylprednisolone, and dexamethasone are administered at body weight-adjusted doses for 6 months. Two methods can be used to establish the vertebral fracture model. One method is creating a bone defect in the lumber vertebrae, with a minimum diameter or width of 5 mm. Another method is compression vertebra model [39].

The review revealed that the vertebral bone defect model is widely preferred for studying vertebral fractures. Nevertheless, this approach has a significant limitation: it creates an artificial cavity rather than a true fracture, which does not accurately reflect clinical scenario. In this cavity, all tissues, including bone, lipids, and blood vessels, are removed, whereas in actual vertebral fractures, these tissues are intermingled. It is crucial to consider whether this model can adequately support research on bone repair. Our previous clinical studies focused on vertebral fractures and refractures in patients with osteoporosis under conditions of unbalanced stress [60]. We tend to believe that the vertebral bone defect model is more appropriate for studying material filling than the vertebral fracture itself. In addition, the osteoporotic vertebral body contains a significant amount of fat that fills the gaps in the cancellous bone, and the fat content continues to increase as bone mass decreases. The relationship between lipids and bone metabolism in the context of osteoporosis remains unclear [61, 62], and studying the microenvironment of the vertebral fracture area using a vertebral bone defect model presents additional difficulties.

**Fig. 1:**
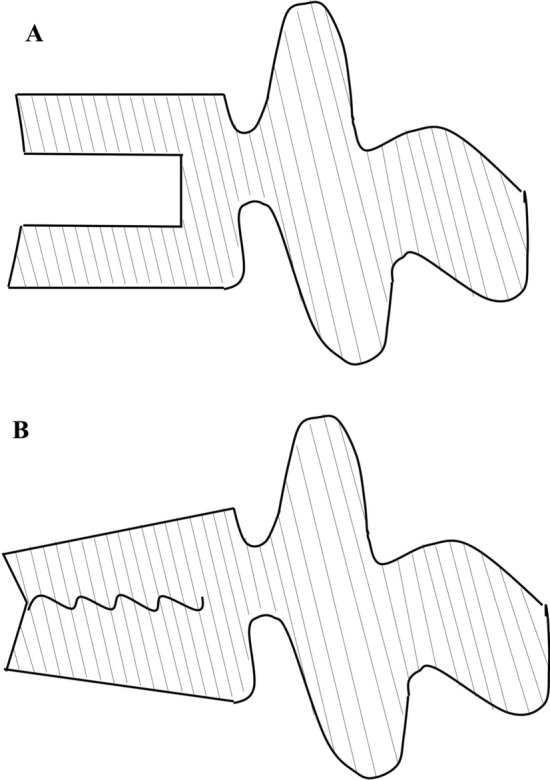
Design scheme for vertebral fracture model. **a** Bone defect scheme. **b** Stress compression scheme

In summary, the literature review indicates that rats, and sheep are the experimental animals that best meet the critical criteria of feeding costs, modeling expenses, and broad applicability. Historically, the methods for modeling vertebral fractures were relatively straightforward, with bone defect models being widely employed in various animal studies. Smaller animals tend to exhibit spontaneous fracture characteristics more readily. However, it is important to note that bone defects do not accurately represent true fractures, a concept acknowledged by numerous researchers. Eschler et al. [39] introduced an innovative compression fracture model that replicates fractures while maintaining the integrity of the bone and surrounding tissue environment. This development may significantly enhance future research on osteoporotic vertebral fractures. Figure 1 illustrates two schematic representations of vertebral schemes.

## Data Availability

No datasets were generated or analysed during the current study.
